# Is There a Role for Spacer Exchange in Two-Stage Exchange Arthroplasty for Periprosthetic Joint Infection?

**DOI:** 10.3390/jcm9092901

**Published:** 2020-09-08

**Authors:** Elie Kozaily, Emanuele Chisari, Javad Parvizi

**Affiliations:** 1 Rothman Orthopaedic Institute at Thomas Jefferson Universit, 125 S 9th Street, Suite 1000, Philadelphia, PA 19107, USA; elie.kozaily@rothmanortho.com (E.K.); Emanuele.chisari@rothmanortho.com (E.C.); 2 Department of Medicine, Indiana University School of Medicine, Indianapolis, IN 46202, USA

**Keywords:** prosthetic joint infection, total knee arthroplasty, total hip arthroplasty, two stage exchange, spacer, spacer exchange, reimplantation

## Abstract

Periprosthetic joint infection (PJI) continues to be one of the most serious complications after hip and knee arthroplasty. The choice of surgical treatment depends on a multitude of factors like chronicity of infection, host factors, and institutional or surgeon experience. Two-stage exchange remains one of the most commonly used technique for chronic PJI in the United States of America. The intended two-stage revision may involve an additional interim procedure where the initial antibiotic cement spacer is removed and a new spacer is inserted. Mostly, the rationale behind spacer exchange is an additional load of local antibiotics before proceeding to reimplantation. There is no conclusive evidence whether a spacer exchange confers additional benefits, yet it delays reimplantation and exposes already fragile patients to the risks and morbidity of an additional surgery.

## 1. Introduction

Periprosthetic Joint Infection (PJI) is a rare complication after total joint arthroplasty (TJA). The risk following a total knee arthroplasty (TKA) varies from 0.5% to 2% with a slightly lower risk for total hip arthroplasty (THA), less than 1% [1,2]. 

Despite some studies reporting declining trend [3,4], PJI will continue to be a prevalent and a serious therapeutic challenge with the projected increase in hip and knee arthroplasty [5].

In addition to anti-microbial therapy, PJI is managed with surgery [6]. The surgical techniques include debridement, antibiotics and implant retention (DAIR), one-stage and two-stage exchange. The choice of the best surgical technique is based on the presentation [7] and chronicity of the infection [8], the host and local factors [9], and the institutional/surgeon experience. 

Two-stage exchange is the most commonly used approach for chronic PJI in the United States [10,11]. It consists of a first stage where the prosthesis is removed and an antibiotic loaded cement spacer is inserted. After usually four to six weeks of systemic antibiotic therapy, the infection is considered to be controlled. After the antibiotic treatment, the patients go on an antibiotic holiday varying from two to eight weeks before proceeding to the second stage to receive a new implant. Of note, some studies argued against the use of antibiotic holiday [12,13].

Two-stage exchange proved to be effective, with a success rate varying between 75% and 98% [11,14,15] in eradicating infections. In addition, joint functionality scores appeared to be improved after completion of the second stage [16,17]. Nonetheless, other studies reported a two-stage related mortality varying between 9% [18] and 25% [19] at a mean follow-up of 69.8 months and 64.8 months, respectively. 

**Figure 1:**
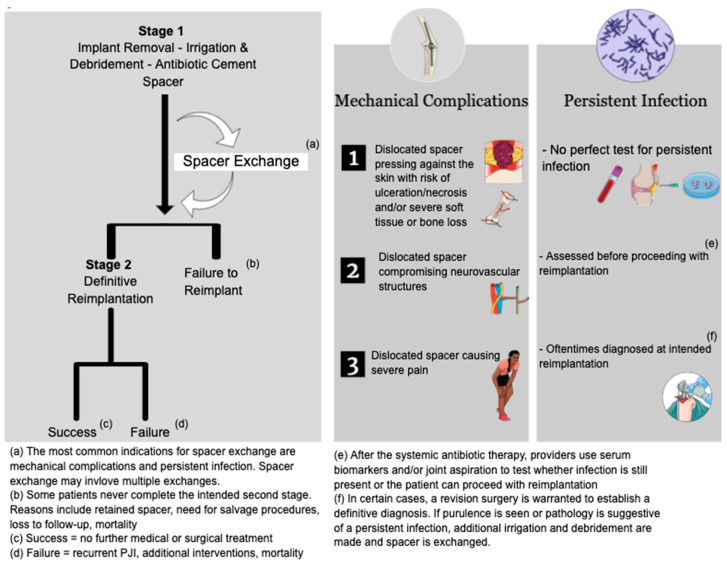
The role of spacer exchange in two-stage exchange and its common indications.

There are circumstances when the initial antibiotic spacer is exchanged in the interim (Figure 1). For instance, a persistent infection, a spacer dislocation or fracture may lead to the additional surgical procedure of ‘spacer exchange’. In the case of a persistent infection, the rationale behind spacer exchange surgery is an interim irrigation, debridement, and the delivery of an additional antimicrobial load through antibiotic impregnated spacers. 

This practice is based on the surgeon’s judgement as well as limited clinical evidence based on helpful, yet not perfect biomarkers. There are no clear guidelines as to whether or when a spacer should be exchanged, hence little is known about the candidates who would benefit most from this procedure. This review aims to highlight the current evidence in order to guide providers in the management of PJI. 

## 2. Spacers

The use of antibiotic impregnated cement spacers allows the delivery of antimicrobials to the soft tissue around the joint. This local concentration is believed to be hard to achieve with systemic antibiotics. 

Despite the efforts and recommendations to adapt antimicrobials used in the spacer to the isolated pathogen [20], vancomycin and aminoglycosides remain the most commonly used antibiotics [21,22]. Vancomycin and aminoglycosides are broad spectrum antibiotics; they exhibit ideal properties of thermal stability and have minimal influence on the mechanical properties of the cement [23,24]. 

Besides targeting the pathogen, antimicrobials may be adapted to patients’ medical conditions, particularly history of allergy [25,26] and renal function [27,28].

Depending on their type, the spacers can provide a certain degree of mobility in the time period between the first and the second stage. 

### Types of Spacers

Static and mobile spacers can be used for the hip and the knee. They are either hand-made by the surgeon or pre-fabricated. For instance, a literature review by Citak et al. [29] reported superior functional outcomes with the use of articulating spacers for hip infection on one hand, and a higher rate of spacer fracture with the use of surgeon-made articulating spacers on the other hand. However, the authors reported no difference in infection eradication or functional outcomes between surgeon-made and preformed spacers.

Another systematic review by Voleti et al. [30] reported no significant difference in reinfection rates between articulating and static spacers used for knee infection (7% vs. 12%; *p* = 0.2). Despite showing that articulating spacers had a significantly increased range of knee motion (101 degrees vs 91 degrees; *p* = 0.0002) after completion of the second stage, the authors reported similar functional scores with articulating and static spacers. Rates of spacer-related complications were low and similar for both types.

In a recent randomized controlled trial, Nahhas et al. [31] compared outcomes in 32 patients receiving static spacers and 36 patients receiving articulating spacers for TKA infection. Articulating spacers provided a significantly greater range of motion 113.0° (95% CI, 108.4° to 117.6°) in the articulating spacer group, compared with 100.2° (95% CI, 94.2° to 106.1°) (*p* = 0.001) and significantly higher Knee Society scores (79.4 compared with 69.8 points; 95% CI, 72.4 to 86.3 and 63.6 to 76.1, respectively; *p* = 0.043) at a mean follow-up of 3.5 years. Static spacers were associated with a longer hospital stay following removal of the infected implant. Of note, static spacers were associated with a greater need for extensile exposure and a higher rate of reoperation. However, these findings were not statistically significant. 

There is no clear consensus on the ideal type of spacer. Most of the time, the choice of the spacer is based on the host (age, functionality) and local factors (bone loss, the extent of the infection, wound condition) as well as the surgeon’s decision. 

Articulating spacers may be used to maintain a certain degree of mobility and allow the limb function to return after definitive reimplantation [32,33]. However, static spacers would be the better option in some scenarios [24,34]. First, in cases where there is a lack of supportive structure around the joint, namely collateral ligaments around the knee or abductor mechanism around the hip, static spacers may be preferred. Second, extensive bone loss can prompt the surgeons to use a static spacer as it requires less fixation and would put the patients at a lower risk of fracture [35]. Lastly, static spacers allow for sufficient rest of an inflamed joint with a poor overlying skin condition.

## 3. Spacer Exchange

From a diagnostic standpoint, the interim spacer exchange procedure allows for repeat microbiology studies including synovial fluid and tissue cultures to investigate a persistent infection. From a therapeutic standpoint, as mentioned earlier, the rationale behind spacer exchange is a repeat irrigation and debridement, and delivery of a local dose of antibiotics through cement, while preserving the space that is left in the joint, thereby facilitating exposure during second stage reimplantation. 

Spacer exchange may need to be performed, prior to definitive reimplantation, because of persistent infection or mechanical complications. Major studies reported a similar prevalence of spacer exchange, around 17% [21,22] in the course of a two-stage exchange procedure. Furthermore, a small percentage of those patients received multiple exchanges. For instance, George et al. [21] reported a 5% prevalence whereas Tan et al. [22] reported a prevalence of 12% of multiple spacer exchanges.

Nevertheless, multiple studies showed that spacers can be colonized by bacteria [35,36]. For instance, Ma et al. [36] reported that bacterial nucleic acids were detected on 54% (n = 7/13) of cement spacers at reimplantation, after a 6-week antibiotic treatment. Yet, none of the patients with detectable bacterial nucleic acids developed recurrent PJI at 15 months follow-up. This highlights the colonization of antibiotic impregnated cement spacers through the presence of highly resistant biofilms. Moreover, Sigmund et al. [37] reported that 17% (n = 17/99) of the patients undergoing two-stage exchange for hip PJI had positive cultures at reimplantation. Sixteen of those patients were considered to have persistent infection, defined by the authors as discharging wound and/or increasing CRP without any other infection focus and/or local signs of infection. Among those, three patients had different microorganisms from the ones isolated at explantation. Thus, the presence of spacers is meant to be a temporary stage.

### 3.1. Reasons for Spacer Exchange

#### 3.1.1. Mechanical Complications

Spacers may be exchanged due to mechanical complications. For instance, Struelens et al. [38] reported a 12% (n = 18/154) incidence of major mechanical complications like spacer dislocation, fracture or subluxation in patients receiving an articulating spacer for periprosthetic knee infection. The authors also reported minor complications like spacer tilting (24%) and mediolateral translation (21%). When it comes to hip spacers, Jung et al. [39] reported a dislocation in 17% (n = 15/88) and spacer fracture in 10.2% (n = 9/88) of the patients. Tan et al. [22] reported that out of 90 patients with spacer exchange in their series, 18% (n = 16/90) were due to mechanical reasons. 

Common underlying reasons for spacer mechanical complications include inadequate soft-tissue tension, incorrect positioning of the spacer, or extensive bone loss [40].

Nonetheless, mechanical complications leading to spacer exchange raise the question whether the use of cement spacers is rather beneficial in the prosthesis-free period, particularly in hip infection. In fact, a major concern is leg length discrepancy which is thoroughly described after resection arthroplasty without the use of spacers. For instance, Diemen et al. [41] reported a prevalence of 8% in a series of 136 hip infections with the use of a cement spacer, with a mean discrepancy of 26 mm; plus, the authors report a reinfection rate of 13% (n = 18/136). In contrast, Sigmund et al. [37] reported a 14% prevalence of leg length discrepancy without the use of cement spacer, with a median discrepancy of 15 mm. In this latter study, the group of patients with a prosthesis-free interval less than 10 weeks had an 8% prevalence of leg length discrepancy with a median of 13 mm compared to a prevalence of 20% and a median of 20 mm in patients with prosthesis-free interval of more than 10 weeks; the authors report a total reinfection rate of 10% (n = 9/93). These numbers suggest that a resection hip arthroplasty with no cement spacer and a shorter prosthesis-free interval may have similar outcomes on leg length discrepancy compared to cases where a spacer is used. However, randomized trials comparing both strategies are lacking. 

The indications to exchange a spacer with a mechanical complication were addressed in the 2018 International Consensus Meeting (ICM) on musculoskeletal infections. With a supermajority consensus [42], the experts agreed that in the event of a mechanical complication, an exchange of the spacer may be done if the spacer (1) is pressing against the skin with an imminent risk of necrosis, (2) is compromising neuro-vascular structures, or (3) is causing pain and disability for the patient. Should other complications occur, namely a dislocation or a fracture, the spacer is safe to leave in place until the definitive second stage surgery.

#### 3.1.2. Persistence of Infection

**Figure 2:**
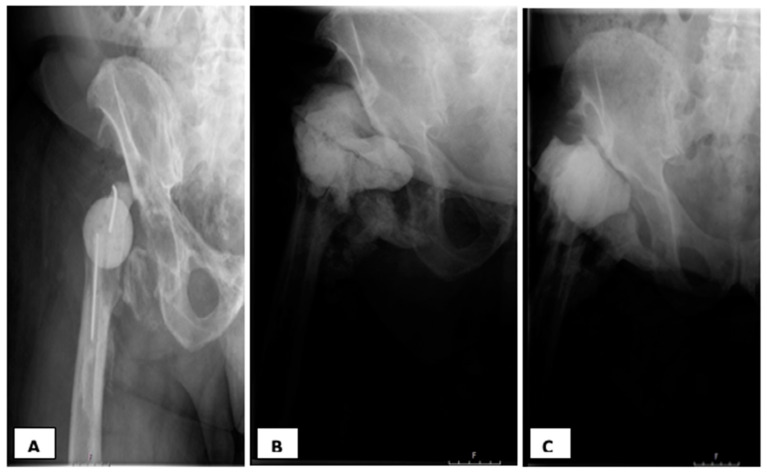
Hip radiographs: periprosthetic joint infection (PJI) with history of three prior debridement surgeries and systemic antibiotics. (**A**) First Hip spacer (Girdlestone); (**B**) Status post second spacer exchange (third spacer) for persistent infection; (**C**) Status post third spacer exchange: failure of intended reimplantation. Clinical history: First exchange occurred after recurrence of fever, elevated CRP, local signs of hip infection and positive urinalysis s/p two-weeks off antibiotics. Of note, purulence was observed during the first exchange surgery. Second Exchange occurred after a purulent drainage, with an aspiration culture growing methicillin-resistant *Staphylococcus aureus* (MRSA). After a third spacer exchange, the intra-operative cultures grew Klebsiella Pneumoniae.

Although persistent infection is the most common indication for spacer exchange, there is no perfect test to confirm it (Figure 2). For instance, Tan et al. [22] reported that the majority (82%; n = 74/90) of spacer exchanges in their series were done for a persistent infection; persistent infection was defined by poor wound healing or intraoperative purulence in addition to high serum inflammatory markers. The patients in the exchange cohort had a higher body mass index (BMI), were more likely to have rheumatoid arthritis, an index revision surgery and a resistant organism. George et al. [21] sought to compare patients with hip and knee infections who underwent a classic two-stage exchange to patients with an interim spacer exchange. The only indication for spacer exchange in their study was a persistent infection. Persistent infection was defined by non-healing wound, ongoing drainage, or pain, in accordance with Delphi criteria for the success/failure of two stage exchange [43]. The authors reported that patients with spacer exchange had significantly more comorbidities (higher Charlson Comorbidity Index- CCI) and a higher prevalence of resistant microorganisms, yet the latter finding did not reach statistical significance (*p* = 0.091). 

The findings in the aforementioned studies imply that the patients who received spacer exchange secondary to persistent infection share common characteristics: multiple comorbidities and more resistant microorganisms.

Multiple studies rushed to investigate histopathology and biomarkers as ways to determine the control of the infection prior to, or during the completion of the second stage.

Findings on histopathology at the moment of reimplantation were reported by a couple of studies. According to Musculoskeletal Infection Society (MSIS) and International Consensus Meeting (ICM) criteria, the presence of five or more Polymorphonuclears (PMNs) per High Power Field (HPF) in operative samples correlates generally with the presence of an infection in a revision arthroplasty [44,45]. However, in a series of 15 patients, Cho et. al [46] reported that the presence of 5 to 20 PMNs per HPF at the moment of reimplantation was not associated with re-infection (n = 0/15) at a minimum follow-up of two years. Moreover, George et al. [47] using a threshold of five PMNs per HPF in at least three HPFs, demonstrated that histopathology was 94% (95% CI, 89–99%) specific yet only 50% (95%CI, 13–88%) sensitive in detecting a persistent infection at reimplantation. In the same study, George et al. reported that MSIS criteria had a high specificity 94% (95% CI, 91–100%) but a poor sensitivity 26% (95% CI, 9–44%) in detecting a persistent infection.

Zmistowski et al. [48] demonstrated that synovial white blood cells (WBC) and PMN prior to reimplantation were higher in patients with persistent infection. In fact, persistent infection was present in 25.6% of patients (n = 33/129) and was defined as a positive aspirate culture, positive intraoperative cultures, or persistent symptoms of PJI, including subsequent PJI-related surgery. Yet, receiver operating curves (ROC) showed poor sensitivity and specificity of WBC and PMN, casting doubt over their utility. In addition, Kusuma et al. [49] reported that synovial fluid WBC was a better marker of a persistent knee infection, two weeks after antibiotic cessation and prior to intended reimplantation. In their study, ESR and CRP performed poorly; they were found to be falsely positive (ESR 54% and CRP 21%) in cases where the infection was considered to be resolved. 

Furthermore, synovial fluid alpha defensin showed poor sensitivity and accuracy in determining control of the infection at the time of reimplantation [50].

After serum D-dimer gained particular interest in the diagnosis of PJI, Shahi et al. [51] investigated its role in determining the time of reimplantation. Of the 29 patients who underwent reimplantation in their cohort, five patients had a high D-dimer. Two of the five patients had a positive culture from intraoperative specimen and subsequently failed the two-stage exchange. Of note, these two patients had falsely negative CRP and ESR values. Nonetheless, a recent study by Wu et al. [52] showed that D-dimer and fibrinogen were of limited value in determining ideal timing for reimplantation. 

Lastly, synovial fluid aspiration in the presence of a spacer, prior to second stage reimplantation, showed poor sensitivity 21% (negative predictive value 51%, specificity 100%, positive predictive value 100%) for the detection of a persistent knee infection [53]. In this latter study, the final diagnosis of a persistent infection was based on intraoperatively acquired cultures and pathology samples during the second stage surgery.

Moreover, as studies failed to agree on an ideal timing for reimplantation, the moment to determine whether the infection is resolved is still unknown. In a retrospective cohort study, Aali-razaie et al. [54] reported that time from resection to reimplantation was not associated with treatment failure (failure was defined by Delphi criteria). Yet, using 26 weeks as a cut-off, the patients with a time to reimplantation >26 weeks had merely significant (*p* = 0.057) higher prevalence, 43.8%, of treatment failure compared to 21.1% in patients with <26 weeks to reimplantation. In a recent retrospective analysis, Sigmund et al. [37] reported that patients with >10 weeks between resection hip arthroplasty and reimplantation had more total complications 66 (n = 49) compared to 80 (n = 44) in patients with a time interval of 10 weeks or less, yet these findings did not reach statistical significance (*p* = 0.068). Of note, the complications at explantation, during resection arthroplasty or at reimplantation were not significantly different, however, patients with >10 week-interval had significantly more complications after reimplantation (70% (n = 31/44) vs. 33% (n = 16/49); *p* = 0.012). Similarly, in a retrospective review of a cohort of 314 infected TKAs treated with two-stage exchange, Sabry et al. [55] found that patients with recurrent infection had a significantly longer time to reimplantation median: 124 days (84–184) vs. 96 (70–161) (*p* = 0.015). Furthermore, in a prospective non-randomized trial, Winkler et al. [56] assigned 38 patients with infected THA and TKA to two groups (10 THA and 9 TKA in each group): short interval (4 weeks or less) vs long time interval (>4 weeks) from resection to reimplantation. Reimplantation was done only if the wound was non-draining and CRP down trending. Resection arthroplasty without a spacer was done for infected THA whereas a static cement spacer was inserted for infected TKA. The mean interval between implant removal and reimplantation was 63 days (range 28–204 days) in patients with a long interval and 17.9 days (range 7–27 days) in patients with a short interval. Yet, the patients in both groups received the same antibiotic regimen for the same duration, a minimum of 12 weeks. Outcomes were comparable as only one patient had a persistent infection in the long interval group compared to none in the short interval group; pain and functional outcomes assessed by patient reported outcomes surveys were similar as well. In contrast, when it comes to multidrug resistant organisms, a long interval to reimplantation appears to be safe and beneficial: Babis et al. [53] reported a 100% success in treating 32 hip resistant infections with a prolonged time to interval period of mean 9.2 months (range, 8–12 months). Of note, authors used long durations of intravenous antibiotic therapy (mean 5.1 weeks, range 4–6 weeks) followed by long-term oral administration (mean 17 weeks, range 12–21 weeks). 

From an infectious standpoint, leaving a spacer in the joint for prolonged periods of time does not seem to confer additional antimicrobial activity. The effect of such strategy is hard to evaluate as it coincides with systemic antibiotic treatment.

In summary, in the absence of solid evidence, the ideal timing to confirm the resolution of the infection and determine reimplantation should rely on the combination of clinical assessment and monitoring of biomarkers. 

### 3.2. Spacer Exchange and Reimplantation

Two-stage exchange remains far from perfect when it comes to definitive reimplantation. In fact, Gomez et al. [57] reported that 17% of patients in their series did not receive reimplantation in the course of a two-stage exchange. Similarly, Wang et al. [58] reported no reimplantation in 18% (n = 111/616) of the patients undergoing an intended two-stage exchange, at a minimum 1 year of follow-up. Of the 111 patients who did not receive reimplantation, 26.1% had spacer retention based on a shared decision with the surgeon, 20.7% had a salvage procedure, and half the patients (53.2%) were judged medically unfit for reimplantation thus having their spacer retained. The authors identified multiple risk factors associated with failure of reimplantation including higher CCI, liver disease, infection by Gram negative organisms, the presence of a sinus tract, prior revision for an aseptic failure, but also spacer exchange after the initial spacer insertion. 

If spacer exchange becomes necessary, the chances of completing the second stage greatly decrease. George et al. [21] reported that 26% of patients with spacer exchange did not proceed to reimplantation compared to 17% of patients without exchange. Eventually, patients with spacer exchange had a significantly longer time to reimplantation with a mean of 218 ± 111 days vs. 127 ± 114 days (*p* < 0.001). Tan et al. [22] reported that 30% of patients (n = 27/90) with spacer exchange did not proceed to reimplantation compared to 18% (n = 81/443) of patients without exchange. Furthermore, the numbers showed that 25% of those who had a spacer exchange for mechanical complications did not receive reimplantation, compared to 31% of patients who had an exchange for a persistent infection. The authors further reported reasons for not proceeding to reimplantation in the exchange cohort. The reasons included, from most to least common: (1) medically unfit to receive reimplantation. (n = 11/27), (2) salvage procedures (fusion n = 5, amputation = 3, girdlestone n = 1) for persistent infection, (3) a shared decision between patient and surgeon to retain the spacer (n = 4), and (4) mortality before proceeding to reimplantation (n = 3). 

However, the authors used propensity score matching to compare patients with and those without spacer exchange, they found no higher odds of failure to undergo reimplantation in patients with spacer exchange (adjusted OR = 1.44, 95%CI 0.80–2.60).

In summary, the available evidence is flawed by an inherent bias linked to the characteristics of the patients in the spacer exchange cohort. These patients tend to have more resistant microorganisms, hence harder to treat infections, poor bone stock and soft tissue integrity, hence a higher incidence of spacer-related mechanical complications. The retrospective nature of these studies makes the data difficult to interpret. Furthermore, there is no gold standard approach in order to compare it with alternative strategies. 

Since most of the spacer exchanges are based on the surgeon’s discretion, with no initial plan to exchange the spacer prior to the intended two-stage, there may be a selection bias in regards to the patients who undergo the procedure. The surgeon might be reluctant to re-implant a patient with multiple comorbidities who has a resistant microorganism if a persistent infection cannot be ruled out. 

### 3.3. Impact of Spacer Exchange on the Success of Two-Stage Revision

#### 3.3.1. Success of Two-Stage Revision

In an attempt to identify predictors of success of a two-stage revision, Mortazavi et al. [56] analyzed prospectively collected data on a series of 117 hips and knees undergoing two-stage exchange. The authors reported that culture negative (OR = 4.5; 95% CI 1.3–15.7), *Methicillin-Resistant Staphylococcus Aureus (MRSA)* (OR = 2.8; 95% CI, 0.8–10.3) and increased reimplantation operative time (OR = 1.01; 95% CI, 1.0–1.03) were found to be predictors of failure. While Mortazavi et al. did not find any association between serum Erythrocyte Sedimentation Rate (ESR) and C-Reactive Protein (CRP) at time of reimplantation and failure of two-stage exchange, Dwyer et al. [59] reported that patients with a preoperative serum ESR > 99 mm/hr had a 1.8-times higher risk of failure. Moreover, Dwyer et al. found that patients with synovial fluid WBC > 60,000 cells/µL and synovial fluid neutrophils >92% were 2.5 and 2.0 times at higher risk of failure, respectively.

In 2013, Diaz-Ledezma et al. [43] used a Delphi based method to define a successful treatment of PJI after a two-stage exchange. Success criteria included (1) infection eradication, characterized by a healed wound without fistula, drainage, or pain, and no infection recurrence caused by the same organism strain; (2) no subsequent surgical intervention for infection after reimplantation surgery; and (3) no occurrence of PJI related mortality (sepsis or necrotizing fasciitis). Yet, the aforementioned criteria were based on outcomes after reimplantation, disregarding the period in the interim and the patients not receiving reimplantation.

These various outcomes in the course of a two-stage exchange underlined the importance of establishing new success/failure criteria, not considering reimplantation as a sole starting point. For instance, by dismissing the patients in the attrition group in the study by Wang et al. [58], the success rate based on Delphi Consensus Criteria was 78.2% at 2 years and 72.6% at 5 years, clearly overestimating the success rate of a two-stage revision.

Therefore, the ICM in 2018 agreed that the starting point should be the first stage where removal of the infected implant and spacer insertion take place. The consensus [60] also recommended the classification of the outcomes of a two-stage exchange into three categories: success, failure due to secondary causes, and failure due to PJI. Success is defined by PJI resolution with no further interventions, failure due to secondary causes is defined by aseptic or septic revisions (including DAIR, excluding amputation, resection arthroplasty and fusion) after 1 year or mortality after 1 year, whereas failure related to PJI, whether directly or indirectly, is defined by aseptic or septic revision within 1 year, salvage procedures (amputation, resection arthroplasty, or arthrodesis), retained spacer, or death within 1 year of initiation of PJI treatment. 

#### 3.3.2. Spacer Exchange and the Success of Two-Stage Revision

Exchanging a spacer can influence the success of a two-stage exchange. In fact, George et al. [21], using Delphi based criteria, reported a lower success rate of 67% (9% CI, 53–77) and 64% (95% CI, 50–75) at 2 years and 5 years, respectively, in the exchange cohort compared to 83% (95%CI, 78–87) and 78% (95% CI, 72–82) in the non-exchange cohort. In the same way, patients in the exchange cohort had a significantly lower infection-free survival (adjusted hazard ratio HR = 1.75 (1.05–2.93) *p* = 0.039).

With a mean follow-up of 5.1 years (range 1.0–16.2), Tan et al. [22] reported a higher reinfection rate in the cohort of patients with exchange (41.3%; n = 26/63) compared to those without spacer exchange (22.1%; n = 80/362). Using Delphi-based criteria, the authors reported a failure rate after reimplantation of 22.10% (n = 80/362), 33.33% (n = 4/12), 43.14% (n = 22/51) in patients who did not have a spacer exchange, patients who had spacer exchange for mechanical complications and patients who had spacer exchange for persistent infection, respectively. This was exemplified by Kaplan-Meier survivorship that showed significantly lower success rate among patients with spacer exchange (*p* < 0.001). Using propensity score matching to compare patients with and those without spacer exchange, the rate of reinfection remained higher in patients with spacer exchange (adjusted OR = 2.23, 95% CI 1.14–4.40).

## 4. Conclusions

Spacer exchange during two-stage exchange is a viable option in patients with a difficult to treat or persistent infection, in accordance with the ICM 2018 recommendations. For instance, in a suspected ongoing infection requiring a revision surgery for diagnosis, exchanging the spacer along with irrigation and debridement is an optimal strategy. Moreover, exchanging the spacer may be an adequate strategy if a persistent infection is diagnosed prior to second stage. These persistent infections usually manifest with continuous drainage and local signs of soft tissue or bone compromise that warrant irrigation and debridement. However, if a persistent infection is diagnosed prior to intended reimplantation and may benefit from prolonged systemic antibiotic therapy, this approach should be adopted. The latter approach spares the patient the multiple risks associated with the additional surgical procedure and the poor outcomes of a spacer exchange in such scenarios.

Nonetheless, in patients with a prior two-stage exchange, and/or a multiple prior spacer exchanges, a special consideration to salvage procedures may be given. These patients likely have multiple comorbidities and more resistant microorganisms. This may be a reflection of the fact that those patients are prone for persistent PJI, which necessitates an interim spacer exchange. The higher failure rates in this population not only elucidate the difficulty of eradicating a resistant infection in a fragile host, but also the morbid effect of an additional surgical procedure prior to reimplantation. A spacer exchange may place the host in a catabolic state and result in additional damage to soft tissues and bone which in turn adversely affects the eventual outcome of PJI treatment.

Furthermore, The ICM 2018 experts agreed with a supermajority that after a failed two-stage exchange, surgeons should consider the patient’s comorbidities and expectations when deciding whether a repeat two-stage will be beneficial for the patient. 

In cases where mechanical complications occur, the ICM 2018 experts agreed—with a super majority—that a spacer may be exchanged in the following scenarios: a dislocated spacer is pressing against the skin with an imminent risk of skin necrosis/ulceration and/or risk of severe soft tissue or bone loss, a dislocated spacer is at risk of compromising neurovascular structures or is causing severe pain.

It is crucial that subsequent studies further address risk factors for failure among patients who receive spacer exchange and investigate biomarkers and predictors of infection control, but more importantly establish reimplantation protocols.

In conclusion, indications for interim spacer exchange should be evaluated fully. If persistence of infection is the reason for interim spacer exchange in patients with multiple comorbidities, a history of multiple surgeries on the affected joint, considerations to salvage procedures may be given.
